# Treatment of Defective Teeth during Pregnancy

**Published:** 1885-03

**Authors:** C. A. Marvin

**Affiliations:** Brooklyn, N. Y.


### Independent Practitioner.

Vol. VI. March, 1885. No. 3.

### Original Communications.

### TREATMENT OF DEFECTIVE TEETH DURING PREGNANCY.

Read Before the Central Dental Association of Northern New Jersey.

BY C. A. MARVIN, D. D. S., BROOKLYN, N. Y.

It may cause a smile of incredulity when I say that the answer not infrequently given to the question embodied in the title of this paper is: "Treat them, as at any other time, with care and thoroughness."

"With care," certainly; "as at any other time," by no means.

I fancy many a dentist can recall instances where the unusual nervousness of a lady patient, under operations not at all formidable nor apparently severe, has surprised and perhaps annoyed him exceedingly. Operations, which before had been borne without a murmur, now cause intense suffering, and the ordinarily quiet, self-possessed patient, whom it was easy and pleasant to work for, has become transformed into a restless sufferer, whose outcries and contortions make a strain upon his sympathies as well as his patience, almost too severe for him to bear.

If this experience occur to him early in his professional life, he finds it puzzling in the extreme. Unable to account for the extraordinary sensitiveness of teeth which he had operated on at other times without difficulty, he concludes, perhaps, that it is all a whim, that his patient is foolishly timid, has lost her self-control, and is making unnecessary trouble. The result of such a conclusion is not favorable to gentleness of speech or of digital exercise. Now, it may be that great injustice is done to the patient by such a conclusion, and that an injury is inflicted as well upon the dentist's eputation. His power of judgment may suffer also, and hence a trinity of evil results proceed from an erroneous opinion.

An intimate knowledge of the lady's real condition of mind and of body might reveal a state of things quite startling to the dentist. For instance, he might discover that her mind was entirely free from whims; that there was a resolution to endure, which, all things considered, was simply heroic; that the tax and drain upon her nervous force were beyond all former measure; that the suffering she manifested was real and of a peculiar acuteness, too much for her quiet endurance, and only to be tolerated through sheer strength of will, never before required of her. While nature shrank, will persisted, and, in reality, the woman was more a heroine twice told than when on former occasions she sat patiently and uncomplainingly through long and complicated operations. Her nervous system was then in repose; it is now in a state of agitation; then she only suffered fatigue, now agony. She is really taxing herself to the utmost to bear, without resistance and without murmuring, the necessary operations. The struggle is terrible, and she will be wearied and limp for hours as a result. No word of harshness or impatience should add to her already heavy burden. She realizes, as no dentist can, what her condition and her experiences are. She can hardly restrain the flood of her tears, both on account of the pain and of her inability to bear it. A woman has a pride of fortitude as well as of beauty, and when it succumbs one may be sure the power to which it bows has no merely imaginary existence. It is real.

If this be her condition, how cruel to add another sting in the form of a severe word or an impatient gesture. Now is the time for the dentist to exercise his magnetic force, if he has any, and, by the steady equipoise of his own nervous energy, influence his patient, gradually reducing her agitation to quietness, or strengthening her by that mysterious transmission of nervous force which, though inexplicable, has been too often realized to be discredited.

So will he be better able to do his work; so will his patient be better able to endure it; so will he learn to exercise an influence, to exert a power which will be of essential service to him all through his professional life; and so will he win the gratitude of the delicate, nervous lady under his hands, and gain a life-long advocate.

But what of this hypersensitive condition of the teeth? And this unusual nervousness of the lady? And the severe struggle which is going on within her, as has been described? What of it all? Why is it?

The patient has entered upon the period of pregnancy, and the period and the state are pregnant of peculiar experiences and phenomenal manifestations.

A wonderful work of Nature is begun. It is the most important, the most trying, the most delicate that the human body is ever called on to undertake. A new living being is to be formed, carried, nourished, and finally, born. In its miniature form it is still to be supplied with all the organs of the mature body—all delicate tissues, all the intricate machinery necessary for the reception and transmission of sensation, for the exercise of functions necessary to the maintenance of life, when that life and the responsibility of preserving it shall have become an independent obligation.

Now, however, this new life is dependent entirely upon the mother. From her it draws its nourishment, from her life its life is sustained, and, as may readily be inferred, the drain upon her is severe. Not alone must she supply the pabulum which the embryonic bony tissues require for their growing solidity, and the muscular tissues for their toughening and development, and the digestive apparatus for its appropriate qualifications for a specific duty, but also the circulatory and nervous systems for their ready action and faithful service. Her nature is tapped, so to speak, in every energy, that the new and dependent life which she carries and nourishes may be supplied. And there is no consideration for her moods or conditions in this new life. Its demands are exacting, imperious, unintermitted. Be she sick or well, cheerful or depressed, fresh or wearied, wakeful or drowsy, the drain is never relaxed, and disregard of its demands is impossible. Her fate is upon her; she can neither escape it nor overcome it. During all the period which nature has fixed for the completion of fœtal life the mother is a slave to her unborn child.

Besides the draft upon the physical energies, there is another as constant and even more taxing, viz.: the drain upon the nervous energy.

If there be a disturbance in any part of the body, notice of its existence and extent is immediately conveyed to the sentiency—the brain's vital tenant—by the alert nerve messengers, which are by no means mere instruments of transmission, or sentinels set to watch and report any damage sustained by any part, but are themselves prime sufferers, and send intelligence to headquarters of their own hurt, as well as of injury to their neighbors. Hence, the report is immediate and reliable. The disturbed part calls for relief, and keeps calling. The system of lines of communication is actively exercised, for the lines are kept busy. This order of affairs is universal and well understood.

Now, take the case we are considering, of the pregnant woman. That there is considerable of a disturbance in a very delicate portion of the body no one will question. And there is good reason for such disturbance. The presence of a mass of matter to which the organ is unaccustomed is no slight thing. It occupies space; it is constantly growing, and, as it grows, it encroaches more and more upon nerves, blood vessels, adjacent organs, displacing, distending, disturbing and making the whole vicinity uncomfortable. Here is a tax upon the strength and the patience which must be experienced to be appreciated. Added to this is the constant feeling of solicitude lest harm should come, through accident, to the burdened woman; and, still further on, an intensity of solicitude, begotten of a new-born love for the living burden, lest harm should come to it, her forming child. What wonder that the woman is wearied with her burden, made uncomfortable by the crowding of every adjacent part by the intruder, worn with constant anxiety? What wonder that the steady disturbance taxes and drains the nervous energy? What wonder if the equipoise of nervous and muscular and digestive systems is deranged or kept in so critical a situation that the slightest untoward influence will derange it?

And if deranged, and these connected systems, delicate each in itself, and composing a marvelously complex machine when united, are out of harmony, the one with the other, what a Pandora's box of ills the pelvic region becomes, while an equally direful condition of things exists in the abdominal and thoracic regions! The avenues of communication with the brain are crowded with complaining suppliants, and the presiding genius is overwhelmed with appeals for help and cries of distress.

So then, this distressful condition of things must be averted, and not invited. And many a female comes into the dentist's hands at a crisis when these two alternatives present themselves for his consideration. If he be unwise in his decision and his manipulation, he invites the sad consequences before described; if he be wise and prudent, he may do much to avert them. A woman in pregnancy should be guarded carefully from disturbing occurrences or disturbing thoughts. Anxiety, apprehension—in a word, *worry* should be kept from assailing her. Her own excited imagination will conjure up enough ills, without their phantom columns being reinforced by real evils. Keep the real ones away, and aid in dispelling the phantoms, if you would do a pregnant woman the greatest service.

Has not enough been said to indicate the kind of treatment the teeth of such a patient should receive at our hands? I will name it temporizing preventive treatment—treatment for the prevention of further damage for the time being. That is just it.

There are cavities possibly in such a mouth, which the dentist could fill permanently and without much pain. Whether it is wise or not to do so will depend largely upon the temperament of the patient. A phlegmatic individual might sit through the necessary appointments and escape all harm, and be loudest in her persuasions to her shrinking sister of a nervous temperament, to pluck up courage and do likewise.

Indeed, the hardships which some women will endure, even when in an advanced stage of pregnancy, and endure them day after day, are simply appalling. And in many such cases also, the offspring does not seem to have been injuriously affected. The child is born in due time, is well formed, hardy and perfect. That such instances are to be found does not weaken the argument before made that, during this period, women should be spared anxiety and nervous excitement. There is a wide difference between nervous excitement and physical or muscular weariness. The women who can bear the hardships without harm, are not the women who are easily susceptible to nervous disturbance. They are strong, hardy, unimaginative, self-reliant women. They may have strong passions, but are not nervous. Or, if they are to some degree susceptible to nervous disturbance, they can still incur muscular weariness without necessarily suffering nervous prostration or excitement. On the contrary, a moderate degree of physical exercise, even to the extent of slight fatigue, is doubtless beneficial both to the mother and her child. But if these same strong, hardy women *should* receive a nervous shock, or experience an over-drain on their nervous energy, the result would be disastrous. It is not easy to produce such a shock on such women; certainly not easy enough for the ordinary and even extraordinary events of common life to effect it. It is not that they are differently made, nor subject to different laws, nor unaffected by the same laws; it is simply harder to touch the key-board of their nervous mechanism. It is encased within non-conducting walls, and the person is, so to speak, iron-clad.

Now, naval officers do not instruct their gunners to open fire upon an iron-clad vessel just because she is iron-clad, and can perhaps stand it without injury. So, unless some extraordinary reason exists for performing at once the dental operations required by such a patient, it seems higher wisdom to hold to the rule and do only temporary work. Cavities sufficiently prepared to receive and retain protecting gutta-percha fillings; gentle manipulation of the mouth through the whole sitting; avoidance of such methods, materials, appliances as seriously irritate the nervous system; a pleasant and hopeful view of the case; an encouraging word as to the condition of the teeth, their safety for months to come, their reduced sensitiveness when ready for permanent fillings—all these details enter into the proper treatment of patient and teeth at such a period.

If it is an important condition of the most successful operations upon the teeth, that the patient—any patient—be in a quiet and equable frame of mind during their performance, as it most certainly is, then every consideration of welfare of patient, excellence of operation and comfort of operator, would emphasize the propriety of performing only temporary dental operations for persons disqualified for preserving equability of mind and equipoise of nerve, by reason of a great demand made upon all their vital forces by nature.

It is the physician's duty to assist nature. It is the dentist's duty also. He can assist her and he can incommode her. He can outrage her. He can mar, perhaps destroy, her beautiful designs.

Neither is it necessary that it be his intention to do so. He can accomplish so evil a result as surely by ignorance, by thoughtlessness. Unwise operations during pregnancy may result in premature birth, or a weak, unbalanced, blighted child, peevish, morbidly timid, dyspeptic in earliest infancy, giving poor promise of brightness in time to come, and this fact is a severer burden upon the mother's heart and hands than even the growing child in fœtal life was to her weary body.

But some one may ask: "If these things are so, would it not be wisest of all to postpone any operation upon the teeth till after the period is over and the patient restored?" I answer, unfortunately, decay will not wait for you. It steadily progresses, and generally progresses more rapidly in such a period. There is a perceptible lowering of tone as well as quickening of sensibility in the dental tissue. The one condition invites attack, even advancing to meet it; the other reduces the ability to resist or endure its injury. So then, if the teeth are neglected there is great danger that some of them may be lost or seriously damaged by the delay. They must have attention, and the kind of attention advocated in this paper seems best calculated to insure the welfare of the teeth, as well as the welfare of the pregnant patient. For the teeth are specially sensitive; they are reduced in tone; there is a loss of vigor; an element of health is wanting; in a word, they are not as able to bear kindly thorough operations and escape the troop of ills that lie in wait for a weak tooth after it has undergone a severe operation.

Sensitiveness to cold, or, what is worse, hot drinks or air, soreness to the touch, elongation, looseness, death of the pulp, ulceration—you know them all, gentlemen; these all, in their order, are waiting to rush in and occupy the territory, if only they are given the opportunity. A good, healthy tooth defies them, when a weak one cannot successfully oppose their entrance. Now a defective tooth in the mouth of a pregnant woman of nervous temperament, is a delicate thing to handle if one would reap good results. Better a hundred times treat it delicately and temporarily than to take a risk so great. Both sides of the argument are in favor of this decision. A temporary filling will preserve the tooth as well as any other for the time being, and it cannot do any harm. Even if the tooth could be treated permanently without danger, no harm is done it by the temporary treatment. The tooth is saved, and can be permanently filled at a future time just as well as now; the patient is relieved both from anxiety and suffering, and everybody is happy.

But another point calls for consideration. Suppose such a patient has teeth with exposed pulps, or with dead and putrefying pulps, or has an alveolar abscess; how in such a case? In the first place, may a kind Providence spare every pregnant woman from such a sad experience. Secondly, if such cases must be, may the same kind Providence spare any of our patients from getting in such a condition. Thirdly, if this cannot be, take the case in hand and treat it as delicately as is possible. Remedies for temporary relief are on the shelves of our medicine cases; use them. If unsuccessful, the best judgment must be exercised, and the two sides of the question carefully weighed. Which will be worse in its effect upon the patient; protracted suffering or such an operation as will be effectual? When consultation is had and a decision is reached, if accepted by the patient, let the dentist proceed to execute it calmly, cheerfully, and with a vivid sense of responsibility, regarding the necessity as not of his creation, and holding it to be his mission mercifully to relieve the sufferer thus thrust into his hands.

Such is wisdom; such is the dentist's duty; such responsibility must he take. In all such cases it is important that the dentist and the physician should consult freely, that the patient may receive the best services as the result of their combined wisdom.

I need only add that in order to make such consultations agreeable and profitable, it is necessary that dentists study carefully and become familiar with this department of physiology, that physicians who are supposed to be thoroughly versed therein may recognize and admit the wisdom of their suggestions, and no time be lost in the endeavor to create confidence or overcome opposition arising from an adverse judgment of the dentist's competency to give advice.

